# Case Report: Early bilateral capsular contraction syndrome associated with Epstein–Barr virus-positive uveitis after cataract surgery

**DOI:** 10.3389/fmed.2026.1901790

**Published:** 2026-08-28

**Authors:** Ying Li, Zhouyang Ye, Minhong Fei

**Affiliations:** 1 Department of Ophthalmology, Zhongnan Hospital of Wuhan University, Wuhan, China; 2 Jiangyan Hospital of Traditional Chinese Medicine, Taizhou, China

**Keywords:** aqueous humor, capsular contraction syndrome, cataract surgery, Epstein–Barr virus, metagenomic next-generation sequencing, viral uveitis

## Abstract

**Objective:**

To report a case of capsular contraction syndrome complicated by viral uveitis during the early postoperative period after bilateral cataract surgery.

**Methods:**

A retrospective case report was conducted.

**Results:**

A 74-year-old woman with no significant medical history developed bilateral capsular contraction syndrome one month after bilateral cataract surgery. Further ophthalmic evaluation revealed bilateral uveitis. Laboratory testing and metagenomic next-generation sequencing identified Epstein–Barr virus (EBV) in both peripheral blood and aqueous humor samples.

**Conclusion:**

EBV-associated uveitis may have contributed to the early development of bilateral capsular contraction syndrome in this patient after cataract surgery. In patients presenting with postoperative uveitis accompanied by capsular contraction, underlying viral infection should be considered as a potential contributing factor.

## Background

Capsular contraction syndrome (CCS) is an uncommon complication after cataract surgery, with an incidence of approximately 1.4% to 14% within 3 to 30 weeks postoperatively (1). It is characterized by progressive centripetal shrinkage of the capsulorhexis opening, accompanied by fibrosis of the anterior lens capsule and a reduction in the effective capsular opening area. CCS may result in intraocular lens (IOL) decentration or tilt, visual impairment, and postoperative refractive changes (2). It usually becomes clinically apparent within the first month after surgery, progresses most rapidly between two and three months post-operatively, and subsequently tends to stabilize. Current evidence indicates that intraoperative parameters (e.g., capsulorhexis size during continuous curvilinear capsulorhexis, and the material and design of intraocular lenses) and patient comorbidities could lead to CCS, including infection, uveitis, high myopia, retinitis pigmentosa, pseudoexfoliation syndrome, diabetes mellitus and myotonic dystrophy. Among these, uveitis represents a heterogeneous group of intraocular inflammatory disorders that can cause structural and functional damage to both the anterior and posterior segments of the eye. In recent years, studies on CCS have mainly focused on IOL-related factors and specific predisposing conditions, such as pseudoexfoliation syndrome; however, reports addressing CCS secondary to uveitis remain relatively limited (3, 4).

Here, we report a patient who developed bilateral CCS during the early postoperative period, one month after bilateral cataract surgery. Comprehensive ocular and systemic evaluations, including fundus angiography and routine hematological testing, after excluding underlying metabolic diseases such as diabetes mellitus, suggested that postoperative uveitis may have contributed to the development of CCS. Further analysis of aqueous humor using metagenomic next-generation sequencing (mNGS) detected Epstein–Barr virus (EBV), supporting a diagnosis of EBV-associated uveitis. This case highlights the potential role of virus-associated intraocular inflammation in the development of CCS and may provide clinical evidence for future investigations into the relationship between different types of uveitis and CCS.

## Case presentation

A 74-year-old woman presented to our hospital with a history of decreased vision in both eyes for more than one month. She had undergone sequential bilateral phacoemulsification with intraocular lens (IOL) implantation at our hospital, with a one-week interval between the two procedures. Her visual acuity improved markedly in both eyes one week after surgery; however, one month postoperatively, she experienced a significant decline in bilateral vision. Apart from a history of cholelithiasis surgery, she had no known systemic diseases, infectious diseases, metabolic diseases, previous ocular disorders, or other surgical history.

Preoperative ophthalmic examination showed a best-corrected visual acuity (BCVA) of 20/50 in the right eye and 20/62 in the left eye. Intraocular pressure (IOP) was within the normal range bilaterally. Slit-lamp examination revealed clear corneas, round pupils, quiet anterior chambers, and cortical lens opacity, with no obvious inflammatory cells. Fundus examination showed no significant abnormalities. The axial length was 22.83 mm in the right eye and 22.75 mm in the left eye. During cataract surgery in both eyes, a continuous curvilinear capsulorhexis (CCC) of approximately 5.5 mm was created. A Johnson & Johnson ICB00 IOL was implanted in the right eye, and a Proming AT3DH IOL was implanted in the left eye. Both procedures were uneventful. Postoperatively, topical dexamethasone eye drops and ointment were administered for anti-inflammatory treatment. One week after cataract surgery, BCVA improved to 20/20 in both eyes, and no significant abnormalities were observed in the anterior chamber or fundus.

**Figure 1:**
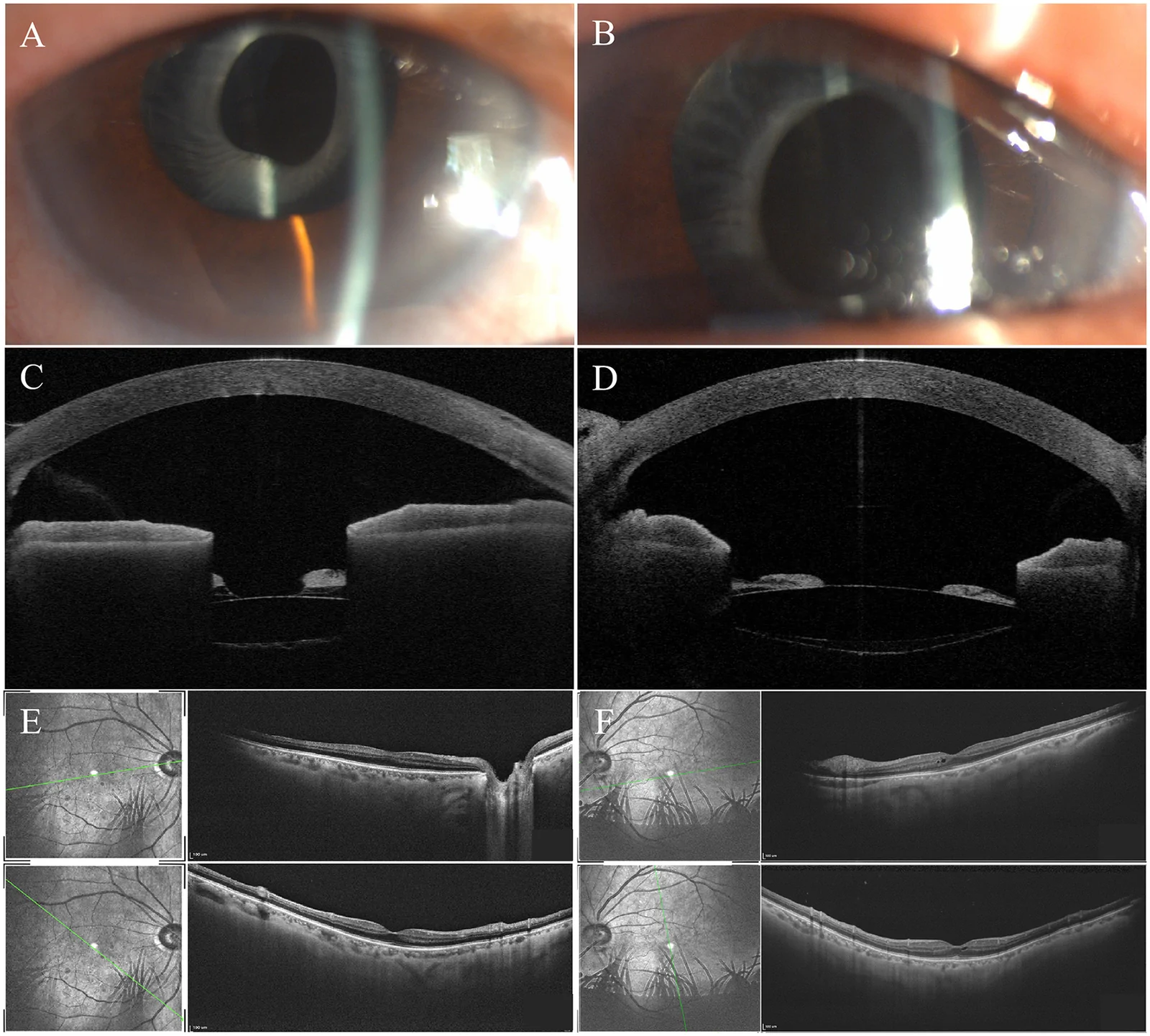
Ocular examination findings at presentation in the patient with capsular contraction syndrome. **(A, B)** Anterior segment photographs of the right and left eyes, respectively, showing capsular contraction and fibrotic membrane formation over the anterior surface of the intraocular lens. **(C, D)** Anterior segment optical coherence tomography (AS-OCT) images of the right and left eyes, respectively, showing increased central corneal thickness (CCT = 648 μm in the right eye, CCT = 662 μm in the left eye) and keratic precipitates on the corneal endothelium. **(E, F)** Swept-source optical coherence tomography (SS-OCT) images of the macula in the right and left eyes, respectively. CCT, central corneal thickness.

At presentation, one month after cataract surgery, BCVA had decreased to 20/40 in the right eye and 20/62 in the left eye. IOP remained within the normal range bilaterally. Slit-lamp examination showed round pupils, mild corneal oedema, anterior chamber flare, and keratic precipitates and well-positioned IOLs in both eyes (Figures 1A–D). A dense white fibrotic proliferative membrane was observed over the central anterior surface of the IOL, and a transparent peripheral proliferative membrane with radial folds was also noted (Figures 1A–D). Further fundus examination revealed macular edema (Figures 1E, F). Fundus fluorescein angiography (FFA) showed a normal arm-to-retina circulation time in the right eye. Late-phase images revealed marked retinal capillary leakage, with fluorescein accumulation in the macular region and optic disc hyperfluorescent staining (Supplementary Figure 1). Based on these findings, the patient was diagnosed with bilateral capsular contraction syndrome (CCS), bilateral uveitis, and macular oedema in the left eye.

Further laboratory testing and metagenomic next-generation sequencing (mNGS) of aqueous humor were performed. Epstein–Barr virus (EBV) DNA was detected in peripheral blood at 8.27 × 10^4^ copies/mL. EBV double-stranded DNA (dsDNA) was also detected in the aqueous humor, with 503 sequence reads, a relative abundance of 68.94%, and a confidence level of 99%. Additional abnormal laboratory findings included elevated white blood cell count, hemoglobin level, absolute neutrophil count, absolute lymphocyte count, and absolute monocyte count.

The patient was treated with systemic and topical ganciclovir as antiviral therapy, together with topical dexamethasone for intraocular inflammation. After stabilization of the virus-associated inflammatory response, she underwent bilateral surgical anterior capsulotomy with membranectomy for CCS to improve visual acuity. The patient subsequently underwent bilateral surgical anterior capsulotomy with membranectomy for capsular contraction syndrome. On postoperative day 1, the contracted capsular membranes in both eyes had been successfully released and excised, restoring a clear visual axis (Supplementary Figures 2A, B). At the one-month follow-up, the visual axes remained clear, with no evidence of recurrent capsular contraction (Supplementary Figures 2C, D). Both corneal edema and macular edema gradually improved after surgery. At one month, anterior segment optical coherence tomography demonstrated a marked reduction in central corneal thickness, while macular optical coherence tomography showed no significant residual retinal thickening or edema (Supplementary Figures 2E, H). Best-corrected visual acuity improved to 20/25 in both eyes. At the two-month follow-up, visual acuity and slit-lamp findings remained stable. The patient remains under regular follow-up at our institution.

## Discussion

This case report retrospectively describes a patient who developed bilateral capsular contraction syndrome (CCS) during the early postoperative period after uneventful bilateral cataract surgery. Based on the clinical findings, laboratory results, and metagenomic next-generation sequencing (mNGS) analysis of aqueous humor, EBV-associated uveitis was considered a potential contributing factor to the development of early-onset CCS in this patient.

The pathogenesis of CCS is generally considered to involve two major mechanisms. First, residual lens epithelial cells (LECs) at the capsulorhexis margin may undergo excessive proliferation, migration, and fibrotic metaplasia in response to various stimuli. These activated LECs can produce extracellular matrix and exert contractile forces, ultimately resulting in progressive shrinkage of the anterior capsular opening. Second, structural or functional weakness of the zonular apparatus may disturb the mechanical balance between the centrifugal force generated by zonular tension and the centripetal force generated by capsular fibrosis and contraction. This imbalance may further promote deformation and contraction of the capsular bag (5). Because intraocular lens type and material have been reported to influence posterior capsule opacification, capsular bag contraction, and postoperative uveitis (4, 6), we also assessed the intraocular lenses implanted in both eyes of the patient. Both lenses were made of hydrophobic acrylic materials. As modern hydrophobic acrylic lenses, ICB00 and Proming intraocular lenses are not generally regarded as high-risk implants for postoperative uveitis or capsular bag contraction, suggesting that the lens material was unlikely to be the primary cause in this case.

Since uveitis can both amplify intraocular inflammation and affect zonular integrity, its potential role in the onset and progression of CCS warrants further attention. Uveitis is a heterogeneous group of intraocular inflammatory disorders with diverse etiologies and clinical subtypes. The differential diagnosis included common infectious causes of uveitis. HSV-1/2, varicella-zoster virus (VZV), and cytomegalovirus (CMV) are well-established causes of viral anterior uveitis, whereas HHV-6 and HHV-7 have been less frequently implicated in intraocular inflammation (7). Toxoplasma gondii is an important cause of infectious posterior uveitis and typically manifests as retinochoroiditis (8). Mycobacterium tuberculosis can cause inflammation involving the anterior, intermediate, or posterior segment, including panuveitis and retinal vasculitis (9). Similarly, Treponema pallidum can produce a wide spectrum of ocular manifestations and should be considered an important infectious mimic in patients with uveitis (10). In the present case, metagenomic next-generation sequencing of the aqueous humor screened for HSV-1, HSV-2, VZV, CMV, HHV-6, HHV-7, T. gondii, M. tuberculosis complex, and T. pallidum. No sequence reads corresponding to these pathogens were detected, providing no mNGS evidence to support these alternative infectious etiologies. Nevertheless, because the sensitivity of aqueous-humor mNGS may be influenced by pathogen burden, sample volume, prior treatment, and the timing of sampling, negative findings cannot completely exclude an infection with a low intraocular pathogen load (11).It is prone to recurrence and may become chronic in some patients, leading to severe ocular complications and irreversible visual impairment. A key pathological feature of uveitis is disruption of the blood–ocular barrier, which promotes the intraocular release of inflammatory cytokines and growth factors. These inflammatory mediators may stimulate residual LECs, enhance extracellular matrix deposition, and accelerate capsular fibrosis and contraction. In addition, chronic or recurrent intraocular inflammation may compromise zonular stability, thereby increasing the susceptibility to CCS (12).

Viral infection is an important cause of infectious anterior uveitis (13). However, CCS secondary to postoperative uveitis has rarely been reported in patients undergoing cataract surgery. In most previously reported cases, CCS developed several months to years after cataract surgery, whereas early-onset CCS within one month has been described mainly in patients with recognized risk factors, such as high myopia, retinitis pigmentosa, and pseudoexfoliation syndrome (14). In the present case, the patient had no prior history of ocular disease or systemic conditions typically associated with CCS. The rapid bilateral development of CCS, together with clinical evidence of uveitis and the detection of EBV DNA in both peripheral blood and aqueous humor, suggests that EBV-associated intraocular inflammation may have contributed to the early onset of CCS. Nevertheless, preoperative biological specimens from the cataract surgery were not preserved, so the onset time of EBV infection (pre- or post-operatively) cannot be clarified. Latent EBV may be reactivated by cataract surgery, inducing uveitis and subsequently CCS. Virus-related testing is not routinely performed before uncomplicated cataract surgery in current clinical practice. In this single case, EBV was detected after the patient developed unusually early capsular contraction accompanied by postoperative uveitis. This observation may provide a potential diagnostic and therapeutic perspective for patients presenting with similar postoperative manifestations. However, no causal relationship can be established on the basis of a single case. Further accumulation and systematic analysis of comparable cases are required, with particular attention to demographic characteristics, medical history, preoperative ocular findings, perioperative factors, and postoperative clinical features. Such evidence may help identify patients at increased risk and inform more comprehensive and individualized preoperative evaluation and postoperative monitoring in future cataract surgery practice.

A similar case was reported by Liu et al. (15), in which a patient with uveitis developed CCS one month after cataract surgery. However, the underlying etiology of uveitis was not further investigated in that report. In contrast, in our case, mNGS analysis of aqueous humor and laboratory testing of peripheral blood identified EBV, providing additional evidence for a possible association between EBV-associated uveitis and early postoperative CCS. A plausible, although unproven, link between EBV-associated uveitis and early capsular contraction may involve amplification of the postoperative inflammatory and fibrotic response. EBV DNA has been detected in the intraocular fluids of selected patients with uveitis, and recent evidence of intraocular immune responses against EBV nuclear antigen 1 further supports a possible association between EBV and ocular immune activation (16–18). However, the detection of EBV in an ocular sample does not necessarily indicate active viral replication or establish a direct pathogenic role. Cataract surgery may induce a sustained inflammatory response within the aqueous humor and activate residual lens epithelial cells (19, 20). Recent transcriptomic analysis of human post-surgical lens epithelium demonstrated activation of inflammatory, TGF-β, Wnt/β-catenin, epithelial–mesenchymal transition, and extracellular-matrix-remodeling pathways, together with increased expression of fibrosis-associated genes, including ACTA2, FN1, and TNC (20). TGF-β-mediated epithelial–mesenchymal transition may promote the differentiation of residual lens epithelial cells into contractile myofibroblast-like cells, resulting in α-smooth muscle actin expression, extracellular matrix deposition, cytoskeletal remodeling, and increased contractile force (21). These changes may generate centripetal forces that progressively narrow the capsulorhexis opening and contract the capsular bag (1, 21). We therefore speculate that EBV-associated intraocular inflammation may have intensified the postoperative inflammatory and fibrotic response, thereby contributing to the unusually early bilateral capsular contraction observed in this patient. Nevertheless, this mechanism remains hypothetical, and EBV detection alone does not establish causality. Given the single-case design, our findings primarily provide a potential diagnostic and therapeutic perspective, suggesting that infectious etiologies may warrant consideration in patients presenting with unusually early bilateral capsular contraction accompanied by otherwise unexplained postoperative uveitis. Nevertheless, because this is a single case report, a direct causal relationship between EBV infection, uveitis, and CCS cannot be definitively established. Further studies are needed to clarify whether virus-associated intraocular inflammation can accelerate capsular fibrosis and contraction after cataract surgery.

To the best of our knowledge, this may be the first reported case of early-onset bilateral CCS potentially associated with EBV-associated uveitis after cataract surgery. This case highlights the need to consider viral infection in patients who develop postoperative uveitis accompanied by rapid capsular contraction. Early recognition of infectious uveitis and timely anti-inflammatory and antiviral treatment may help stabilize intraocular inflammation and guide the subsequent management of CCS.

## Conclusion

This report describes a case of bilateral early-onset capsular contraction syndrome (CCS) potentially associated with Epstein–Barr virus (EBV)-associated uveitis after cataract surgery. The findings highlight a possible relationship between virus-associated intraocular inflammation and the rapid development of CCS in the early postoperative period. Therefore, in patients who develop postoperative uveitis accompanied by CCS after cataract surgery, underlying viral infection should be considered. Further studies are warranted to clarify the role of viral inflammation in the pathogenesis and progression of CCS.

## Data Availability

The original contributions presented in the study are included in the article/Supplementary material, further inquiries can be directed to the corresponding author.

## Supplementary material

Supplementary Figure 1 FFA of the case admitted with CCS. **(A)**: FFA of the right eye; **(B)**: FFA of the left eye.

Supplementary Figure 2 Ocular findings during postoperative follow-up. Anterior segment photographs at 1 day and 1 month (**A–D**: **A** and **C**, right eye; **B** and **D**, left eye), anterior segment OCT at 1 month (**E**, CCT = 605 μm in the right eye; **F**, CCT = 614 μm in the left eye), and macular OCT at 1 month (**G**, right eye; **H**, left eye) after anterior capsulotomy with membranectomy.
